# Student Dietitians' Perceptions and Experiences of Objective Structured Clinical Examination to Assess Communication Skills

**DOI:** 10.1111/jhn.70136

**Published:** 2025-10-22

**Authors:** Annemarie Knight, Kevin Walsh, Dianne P. Reidlinger, Patricia Thomas‐Owolabi, Kevin Whelan

**Affiliations:** ^1^ Department of Nutritional Sciences King's College London London UK; ^2^ Faculty of Health Sciences and Medicine Bond University Robina Queensland Australia

**Keywords:** assessment, communication skills, dietetic education, OSCE, simulation

## Abstract

**Introduction:**

Communication skills are essential to dietetic practice and are therefore embedded in dietetics curricula worldwide. Objective structured clinical examinations (OSCEs) are often used to teach and assess communication skills, yet student dietitians' experiences remain underexplored. The aim of this study was to evaluate student dietitians' perceptions and experiences of OSCEs to assess communication skills.

**Methods:**

Student dietitians participated in OSCEs for the assessment of communication skills before the first of two substantial periods of practice‐based learning. These consisted of two or three OSCE stations reflecting in‐patient and out‐patient dietetic scenarios. A questionnaire survey measuring students' perceptions and experiences of the OSCE was conducted. The survey measured five domains: (1) preparedness; (2) environment and processes; (3) fairness; (4) authenticity; and (5) assessment for learning, with responses recorded on a five‐point Likert scale (strongly agree to strongly disagree).

**Results:**

The complete case analysis consisted of 130 student dietitians over four academic years. In general student dietitians had highly positive experiences of the OSCEs, particularly in relation to *assessment of learning* (83.3% positive experience score) and *authenticity* (80% positive experience score). Fairness resulted in the lowest positive experience score (50%). Younger students reported lower positive experiences of *preparedness* (60%) compared with older students (80%) (*p* = 0.045).

**Conclusions:**

Student dietitians have generally positive perceptions and experiences of OSCEs for the assessment of communication skills, but this is not universal for all domains of their experience. There is a need for strategies that enhance preparedness and reduce anxiety in relation to OSCEs in dietetics.

## Summary

- Objective structured clinical examinations (OSCEs) are often used to teach and assess communication skills, yet there is limited research on the perceptions and experiences of student dietitians' regarding this approach.
- Student dietitians reported highly positive experiences of OSCEs to teach and assess communication skills, particularly in relation to ‘assessment of learning’ and ‘authenticity’, although they were less positively rated for ‘fairness’.
- Younger student dietitians reported less positive experiences for ‘preparedness’ for OSCEs.

## 1 Introduction

Objective structured clinical examinations (OSCEs) were developed in the 1970s to assess clinical competence in medical education [1]. Since then, OSCEs have been adopted across various health professions to assess clinical and professional skills [2]. They have been defined as ‘*an assessment tool based on the principles of objectivity and standardisation, in which the candidates move through a series of time‐limited stations in a circuit for the purposes of assessment of professional performance in a simulated environment. At each station candidates are assessed and marked against standardised scoring rubrics by trained assessors’* [2]. Aligned with Miller's pyramid of clinical competence, OSCEs enable students to demonstrate the ability to ‘show how’ rather than simply ‘know how’ [2, 3]. When scenarios are realistic and feedback is of high quality, OSCEs can also enhance learning and contribute positively to educational outcomes [4]. Whilst OSCEs offer considerable benefits such as feasibility, reliability and flexibility, they are also resource intensive and costly [5].

The use of OSCEs as an educational strategy has increased in dietetic education. A systematic review of assessment practices in dietetics from 2017 to 2024 identified 12 of the 59 studies specifically used OSCEs as an assessment method [6]. Their use in dietetic education spans the teaching and assessment of communication skills (e.g. active listening, questioning styles, non‐verbal) and clinical skills (e.g. clinical reasoning, nutritional assessment) and they can help bridge the gap between theory and practice [7, 8]. Performance in an OSCE has been positively associated with both student dietitians' perceived readiness for practice placement [9] and subsequent placement outcome [10]. The OSCE format enables the assessment of a diverse range of dietetic skills including clinical and communication skills [9]; dietary assessment and anthropometry [11]; malnutrition diagnosis [12]; dietary assessment, dietary modification, enteral feeding [10, 13]; preventive health [14]; and dietary assessment, dietary modification, communication skills [15]. Despite this range of published studies of OSCEs in dietetic education, few evaluate student perception and experiences, and where they do, this is frequently limited to brief questions on overall satisfaction.

A significant role is played by OSCEs in the assessment of communication skills. In medical education, OSCEs are widely employed to assess communication skills [16] with research focusing on the validity and reliability of measurement tools over the experience of students [17, 18]. Effective communication is a core component of dietetic practice [19, 20]; consequently, communication skills are featured prominently in dietetic curricula and professional standards internationally [21]. Communication skills teaching in dietetics has shifted from didactic classroom approaches to simulation‐based learning opportunities, such as standardised scenarios for dietitian‐patient communication with a patient actor. However, there is less evidence regarding how communication skills are assessed as students progress through dietetic education [22]. For example, a scoping review of teaching and assessment methods for communication skills in dietetics found that of the 45 studies included, 13 described simulation‐based approaches to teaching, but only two reported using OSCEs in a simulated environment to formally assess communication skills [22]. In summary, OSCEs in dietetics are commonly used to both teach and assess communication skills, either alone or in conjunction with other professional and clinical skills, but there is less literature on how students learn from and experience them.

Evidence has examined the perceptions and experiences of OSCEs as an assessment method among students from many other healthcare disciplines. A systematic review found that nursing students needed adequate time to prepare for OSCEs, experienced anxiety, trepidation and uncertainty related to the assessment, but found OSCEs a valuable learning experience that informed their practice [23]. Similar findings have been reported for students in medicine and pharmacy [24, 25, 26]. However, there has been little examination of how student dietitians experience this assessment leaving educators uncertain about how to best prepare them for OSCEs and understand their engagement with this assessment format.

Understanding student dietitians' perspectives on an OSCE to assess communication skills could inform strategies to maximise the learning benefits of this assessment format and enhance acceptability for students. The aim of this study was to explore student dietitians' perceptions and experiences of an OSCE designed to assess communication skills.

## 2 Methods

This study employed a repeated cross‐sectional design involving the administration of a questionnaire to measure student dietitians' perceptions and experiences following an OSCE to assess their communication skills.

### 2.1 Communication Skills OSCE Assessment

Students enrolled in a Bachelor of Science in Nutrition and Dietetics (4 years), Postgraduate Diploma (1.5 years) or Master of Science in Dietetics (2 years) who completed the communication skills module with the embedded OSCE assessment as part of their pre‐registration education were eligible to take part in this study. Pre‐registration education is the academic and clinical training required to be eligible to practice as a registered dietitian. The module is designed to develop core communication skills required for patient‐centred dietetic practice [19]. It employs an experiential learning model combining taught content (e.g., active listening, empathy, structuring consultations, and explaining dietary information) with communication skills practice using simulated patients (trained patient actors) and facilitated feedback [27]. Students are assessed on these skills in a summative OSCE, a high stakes assessment linked to progression to practice placement during the study period. The module takes place in the latter part of the programmes following the completion of a 2‐week introductory practice placement and before a 12‐week practice placement which is the second of three practice placements integral to the programme.

The OSCE took place in the university clinical simulation facilities and both inpatient hospital ward and outpatient clinic environments were created for the OSCE stations. Before the summative OSCE, students had skills sessions or a practice OSCE in the clinical simulation facility to become familiar with the environment and the assessment format. Consistent with our ongoing evaluation and action learning approach to module delivery adaptations were made to the module each year based on module team reflections and student feedback. These included reducing the number of stations from three to two; providing instant feedback on the day; changing the assessment from a high stakes assessment with a mark awarded to a hurdle assessment which students needed to pass but with no mark awarded. A number of standardised case studies were developed that included a short introduction to a clinical case for students, a detailed role brief for patient actors, and standardised marking criteria for assessors (Box 1). Scenarios included an outpatient with newly diagnosed type 2 diabetes, an outpatient with stage 3 chronic kidney disease requiring low potassium dietary advice, and an inpatient with chronic obstructive pulmonary disease with malnutrition. These were developed to reflect common scenarios during the subsequent practice placement and designed to be consistent in terms of the complexity and communication skills required. Depending on the year of study, students completed two or three OSCE stations with each station based on a different clinical case. Students were provided with details of the clinical case the day before the OSCE so they could prepare and gather any resources they wished to use, for example, preparing explanations of key terms, plan the interview and collate nutrition education resources for the clinical condition. On the day, at each of the two or three OSCE stations they completed a 15‐min interview with a simulated patient (patient actor). Each station had two assessors who were experienced dietetic practitioners or academic dietitians. Assessors underwent training to ensure consistency in marking and feedback. A standardised rubric was used that was developed by the module team and was constructively aligned with the learning outcomes of the module and the communication competencies expected. At the end of each OSCE station, the assessors agreed a mark for the student and provided written feedback on areas of both good practice and areas for communication skill development.

Box 1 **Summary of an illustrative example clinical case study for an OSCE station**

**Communicating with a patient with type 2 diabetes**

**Context**: You are on a practice placement and are having a second consultation with a patient. Last time you discussed their diagnosis of type 2 diabetes and explained the condition. You collected the information provided below, explained the health risks associated with type 2 diabetes and the link between type 2 diabetes and diet.

**Information provided to the student regarding this patient**: Age, weight history and height, living arrangements, employment, lifestyle, diet history, background to diagnosis of type 2 diabetes, medications, and HbA1c.

**At the second consultation:** The patient explains that they find making changes difficult due to money concerns and also thinks that some of the suggestions provided by her GP e.g. giving up sugar are unappealing.

Your supervisor has asked you to complete the following stages of the second consultation:

Greeting and briefly summarise the previous consultation;

Comparing the patient's actual diet with the recommended diet for this condition;

Assessing the patient's readiness for change;

Summarising the information shared in the consultation and closing.

### 2.2 Questionnaire to Evaluate OSCE

Following a literature search, we were unable to identify a previously validated questionnaire to measure OSCE perceptions and experience. In view of this, a bespoke questionnaire was developed specifically for this study by two dietitians with qualifications and experience in higher education research. The questions were developed by reviewing previous studies evaluating perceptions or experiences of OSCEs that identified issues relating to preparedness, environment, fairness, authenticity and learning, and questions were formed under each of these domains from those previous studies [24, 26, 28, 29, 30]. The draft, and final, questionnaire consisted of 28 questions posed as statements with a five‐point Likert scale from ‘strongly agree’ to ‘strongly disagree’.

The questions explored student's perceptions and experiences of five domains related to the OSCE assessment:

1. Preparation for the OSCE (five questions);
2. Conduciveness of the OSCE environment and processes (seven questions);
3. Fairness of the OSCE assessment (four questions);
4. Authenticity of the OSCE assessment (five questions); and
5. OSCE as a learning opportunity (seven questions).

Questions related to each domain were randomly distributed across the questionnaire and only grouped by domain for analysis.

The questionnaire was evaluated for face validity by the academic team involved in OSCE delivery and evaluation design with minor changes to format and wording. The questionnaire did not undergo formal validation.'

All students were invited to complete the questionnaire in paper format either immediately after or within 16 weeks of completing the assessment (the latter for 1 year only due to staff changes). The survey was completed in the academic years 2012–13, 2013–14, 2015–16, and 2017–18 but not in 2014–15 and 2016–17 for operational reasons. This post‐assessment survey was deemed a service evaluation and therefore did not require ethical approval. Participation was voluntary and anonymous.

### 2.3 Statistical Analysis

A “positive experience score” was calculated for each student for each of the five domains. For example, responding ‘agree’ or ‘strongly agree’ for a positively phrased statement (e.g. ‘Feeback after the OSCE was helpful’) or ‘disagree’ or ‘strongly disagree’ for negatively phrased statements (e.g. ‘I felt poorly prepared for the OSCE’) was considered a positive attitude or experience. Each positive response contributed one point towards positive experience score for that domain. A sum score for each domain was calculated and presented as a percentage of the maximum (total number of questions for that domain). Question 22 (‘Feedback after the OSCE was helpful’) was excluded from the calculation for the *Assessment for Learning* domain, as many students had not yet received feedback at the time the questionnaire was completed. For the purposes of calculating domain scores, missing data were considered missing at random and a conservative approach to imputation was followed by coding as neutral.

Data were analysed using IBM SPSS Statistics, Version 29.0.1.0. Descriptive statistics including frequencies and percentages and median [IQR], were used to summarise the data. Distributions of continuous variables were evaluated using histograms and the Kolomorov‐Smirnov test. Categorical variables were compared using Chi‐square tests (e.g. demographic and educational characteristics of undergraduate vs postgraduate students). Non‐normally distributed continuous variables were analysed using Mann‐Whitney U tests (for two groups), or Kruskal‐Wallis tests (for multiple independent groups) with post hoc pairwise significance assessed using Dunn's test. Paired continuous data (e.g. responses from the same student across the five different domains) were analysed using Friedman's Two‐Way ANOVA with post hoc pairwise significance determined by the Wilcoxon rank sum test. Pairwise *p* values have been Bonferroni‐adjusted for correction for multiple testing.

## 3 Results

### 3.1 Participant Characteristics

**Figure 1:**
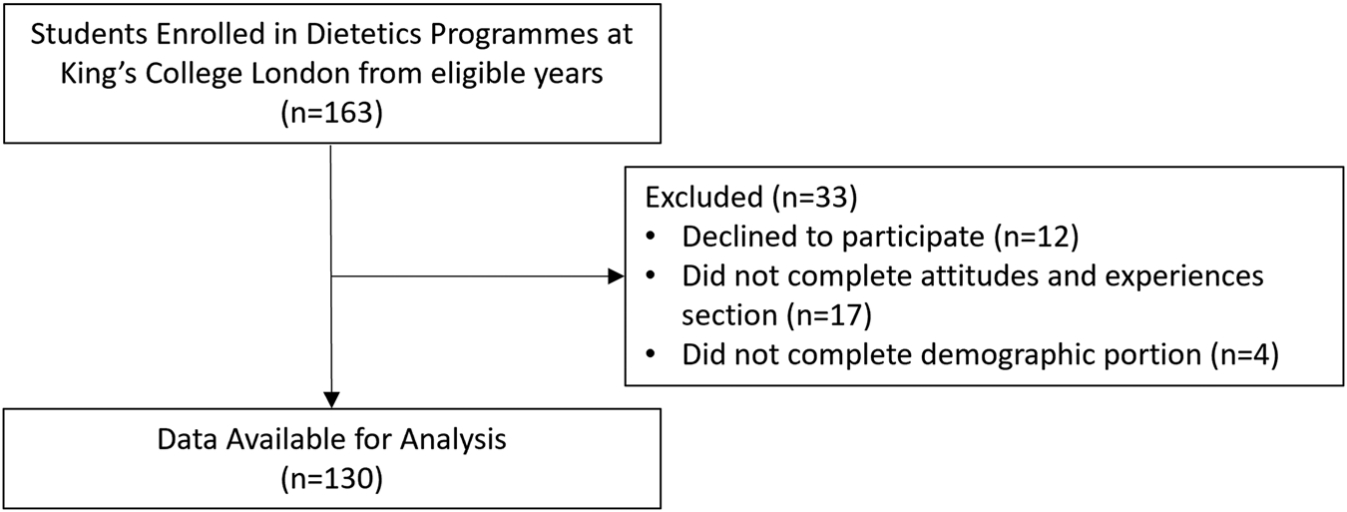
Participant flow diagram.

A total of 163 students were invited to complete the questionnaire, of whom 12 declined to take part, 17 completed only the demographic section of the questionnaire and four did not complete the demographic section, leaving data from 130 students remaining for the complete case analysis (Figure 1).

Participant characteristics for the 130 students are summarised in Table 1, of whom 74 (56.9%) were studying on the BSc Nutrition & Dietetics programme, and 56 (43.1%) on the PG Diploma/MSc Dietetics programme. While MSc students were older (median age 23.6 [3.5] years) compared with BSc students (median age 21.9 [8.0] years, *p* = 0.005), they were similarly distributed between age categories (20–24 y, 25–29 y and 30+ years, *p* = 0.173). Ninety percent of respondents were female, and there was a relatively higher proportion of females studying the BSc (70/74, 94.6%) compared with PG Diploma/MSc (47/56, 83.9%, *p* = 0.045) (Table 1).

**Table 1:** Participant characteristics of 130 student dietitians undertaking the evaluation of attitudes and experiences of OSCEs.

Characteristic	All (*n* = 130)	BSc (*n* = 74)	MSc (*n* = 56)	*p* value*
Age (years), median [IQR]	23.3 [5.4]	21.9 [8.0]	23.6 [3.5]	**0.005**
Age category, *n* (%)				
20–24 years	86 (66.2)	48 (64.9)	38 (67.9)	0.173
25–29 years	21 (16.2)	9 (12.2)	12 (21.4)	
30+ years	21 (16.2)	15 (20.3)	6 (10.7)	
Unspecified	2 (1.5)	2 (2.7)	0 (0.0)	
Gender, *n* (%)				
Female	117 (90.0)	70 (94.6)	47 (83.9)	**0.045**
Male	13 (10.0)	4 (5.4)	9 (16.1)	
Participants by academic year, *n* (%)				
2012–13	34 (26.2)	18 (24.3)	16 (28.6)	0.269
2013–14	41 (31.5)	26 (35.1)	15 (26.8)	
2015–16	24 (18.5)	10 (13.5)	14 (25.0)	
2017–18	31 (23.8)	20 (27.0)	11 (19.6)	
Highest level of qualification, *n* (%)				
A‐level or equivalent	48 (36.9)	48 (64.9)	0 (0.0)	**< 0.001**
Access/foundation course	6 (4.6)	6 (8.1)	0 (0.0)	
Bachelor's degree (BA, BSc)	54 (41.5)	17 (23.0)	37 (66.1)	
Master's degree (MA, MSc)	20 (15.4)	3 (4.1)	17 (30.4)	
PhD	2 (1.5)	0 (0.0)	2 (3.6)	
Previous healthcare experience, *n* (%)	49 (37.7)	26 (35.1)	23 (41.1)	0.439
English as a first language, *n* (%)	119 (91.5)	67 (91.8)	52 (92.9)	0.545

* *p* values are the result of a *χ* ^2^ Test on categorical variables and Mann‐Whitney *U* Test on continuous variables.

The largest numbers of respondents were from the 2013–2014 cohort (41/130, 31.5%), however, there was no significant difference in response rates by cohort (*p* = 0.269). Similar proportions of students in each group reported previous healthcare experience (BSc 35.1%, MSc 41.1%, *p* = 0.439) and having English as a first language (BSc 91.8%, MSc 92.9%, *p* = 0.545). Inevitably, MSc students were more likely to already hold a Bachelors or Masters degree as their highest qualification compared with BSc students (*p* < 0.001) (Table 1).

### 3.2 Student Experience of OSCEs

**Figure 2:**
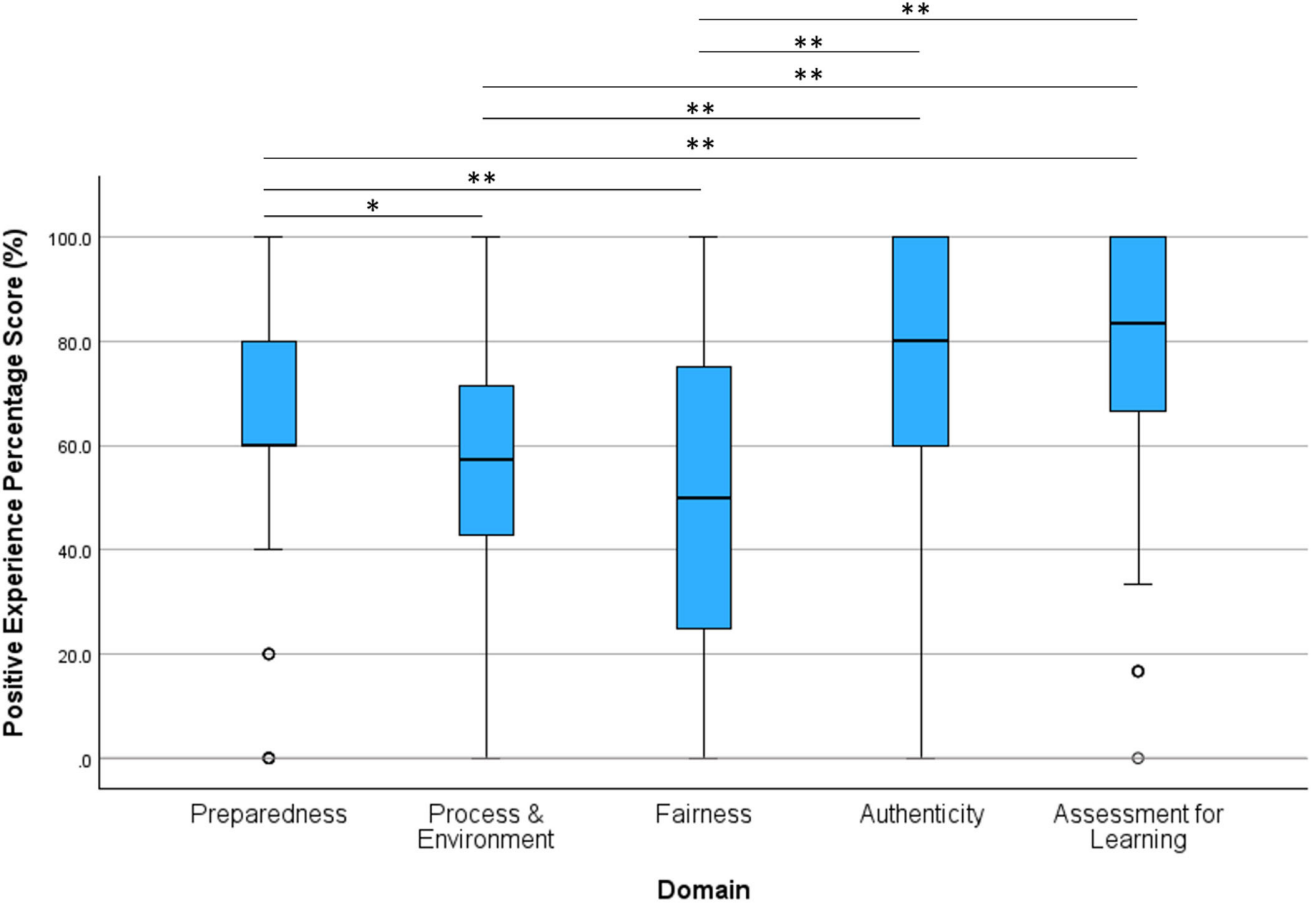
Boxplot displaying percentage scores for positive experiences, organised by domain. Positive scores were compared across domains by Related Samples Friedman's Two‐Way Analysis of Variance by Ranks with an overall test *p* < 0.001. *Post hoc* pairwise comparison between each pair of domains was then performed using a Wilcoxon rank sum test, adjusted for multiple testing using a Bonferroni correction, and significance denoted by *p < 0.05 and ** < 0.001.

Responses to each statement, organised by each of the five domains, are provided in Table 2, and summarised graphically in Figure 2 which compares the positive experience score between each domain.

**Table 2:** Participant responses from student dietitians to statements regarding their attitudes towards, and experiences of, OSCEs, and positive experience scores, organised by each of five domains.

Domain	Question (*n* = 130 unless otherwise specified)	Strongly disagree, *n* (%)	Disagree, *n* (%)	Neutral, *n* (%)	Agree, *n* (%)	Strongly agree, *n* (%)	Positive experience, *n* (%)^b^
Preparedness	2. The tasks at the OSCE stations reflected the skills taught in our communication module (*n* = 129)	0 (0.0)	12 (9.3)	19 (14.7)	74 (57.4)	24 (18.6)	98 (76.0%)
	6. The facilitators in the simulated patient teaching sessions helped me to prepare for the OSCE	3 (2.3)	8 (6.2)	14 (10.8)	70 (53.8)	35 (26.9)	105 (80.8%)
	19. The OSCE took a lot of preparation time	0 (0.0)	37 (28.5)	30 (23.1)	53 (40.8)	10 (7.7)	37 (28.5%)
	20. I felt poorly prepared for the OSCE	8 (6.2)	69 (53.1)	20 (15.4)	27 (20.8)	6 (4.6)	77 (59.2%)
	28. The simulated patient teaching sessions were good preparation for the OSCE (*n* = 128)	4 (3.1)	7 (5.5)	8 (6.3)	66 (51.6)	43 (33.6)	109 (85.2%)
Process & environment	3. I needed more time at each OSCE station	7 (5.4)	60 (46.2)	27 (20.8)	27 (20.8)	9 (6.9)	67 (51.5%)
	8. I found the OSCE to be intimidating	1 (0.8)	28 (21.5)	18 (13.8)	51 (39.2)	32 (24.6)	29 (22.3%)
	11. Instructions at each OSCE station were clear and unambiguous	3 (2.3)	13 (10.0)	19 (14.6)	79 (60.8)	16 (12.3)	95 (73.1%)
	13. The OSCE was well organised (scheduling of students to stations, rooms, times)	0 (0.0)	2 (1.5)	14 (10.8)	74 (56.9)	40 (30.8)	114 (87.7%)
	17. The patient actors at the OSCE stations negatively affected my performance (*n* = 129)	17 (13.2)	53 (41.1)	31 (24.0)	21 (16.3)	7 (5.4)	70 (54.3%)
	23. The environment for the OSCE stations was distracting	9 (6.9)	65 (50)	21 (16.2)	23 (17.7)	12 (9.2)	74 (56.9%)
	25. This OSCE provoked more anxiety than any other examination	9 (6.9)	49 (37.7)	26 (20.0)	27 (20.8)	19 (14.6)	58 (44.6%)
Fairness	1. I thought the OSCE was an accurate assessment of my communication skills	5 (3.8)	24 (18.5)	27 (20.8)	67 (51.5)	7 (5.4)	74 (56.9%)
	5. I thought the OSCE minimised my chances of failing	10 (7.7)	27 (20.8)	57 (43.8)	32 (24.6)	4 (3.1)	36 (27.7%)
	14. The standardised scoring system used by the assessors for the OSCE was fair (*n* = 122)	3 (2.5)	7 (5.7)	54 (44.3)	52 (42.6)	6 (4.9)	58 (47.5%)
	16. Individual student characteristics (e.g., student's personality, ethnicity, gender) do not affect OSCE marks (*n* = 126)	7 (5.6)	23 (18.3)	28 (22.2)	44 (34.9)	24 (19)	68 (54%)
Authenticity	4. I think that OSCE exam marks provide an accurate measure of the communication skills required in dietetics	6 (4.6)	11 (8.5)	20 (15.4)	63 (48.5)	30 (23.1)	93 (71.5%)
	10. The setting and context at each OSCE station felt authentic to clinical practice	9 (6.9)	26 (20.0)	17 (13.1)	64 (49.2)	14 (10.8)	78 (60%)
	15. The OSCE is a practical and useful experience for students	2 (1.5)	6 (4.6)	11 (8.5)	72 (55.4)	39 (30.0)	111 (85.4%)
	18. The OSCE has made me feel more prepared for placement	6 (4.6)	15 (11.5)	16 (12.3)	66 (50.8)	27 (20.8)	93 (71.5%)
	24. OSCEs will help students deal with stressful situations on placement	9 (6.9)	17 (13.1)	15 (11.5)	76 (58.5)	13 (10.0)	89 (68.5%)
Assessment for learning	7. The OSCE stations highlighted my areas of weakness in communication	4 (3.1)	11 (8.5)	26 (20.0)	68 (52.3)	21 (16.2)	89 (68.5%)
	9. The OSCE allowed me to demonstrate a wide range of communication skills	5 (3.8)	11 (8.5)	26 (20.0)	75 (57.7)	13 (10.0)	88 (67.7%)
	12. The OSCE provided me with opportunities to learn (*n* = 128)	4 (3.1)	7 (5.5)	14 (10.9)	78 (60.9)	25 (19.5)	103 (80.5%)
	21. Other skills could be evaluated using an OSCE format	2 (1.5)	19 (14.6)	27 (20.8)	65 (50.0)	17 (13.1)	82 (63.1%)
	22. Feedback after the OSCE was helpful (*n* = 109)^a^	7 (6.4)	16 (14.7)	43 (39.4)	35 (32.1)	8 (7.3)	43 (39.4%)
	26. A written examination would have been a better way to test communication skills (*n* = 129)	73 (56.6)	47 (36.4)	7 (5.4)	2 (1.6)	0 (0.0)	120 (93%)
	27. Having an OSCE format motivated me to learn communication skills	3 (2.3)	4 (3.1)	13 (10.0)	85 (65.4)	25 (19.2)	110 (84.6%)

^a^ Question 22 was excluded from the Assessment for Learning domain score and percentage as 21 students had not received feedback at the time of receiving the questionnaire, therefore the maximum score used to calculate the percentage score for that domain is 6 not 7.

^b^ Positive experience is the total number (%) of students who either agreed or strongly agreed with positively phrased statements (1, 2, 5, 6, 7, 9, 10, 11, 12, 13, 14, 15, 16, 18, 21, 22, 24, 27, 28), or disagreed or strongly disagreed with negatively phrased statements (3, 8, 17, 19, 20, 23, 25, 26).

For the *Preparedness* domain, the median positive experience score was 60.0% (3/5) [IQR 20.0%] with the majority agreeing the tasks at the OSCE stations reflected the skills taught in the communication module (57.4% agreed, 18.6% strongly agreed). However, 33 (25.4%) students felt poorly prepared for the OSCE.

For the *Process & Environment* domain, the median positive experience score was 57.1% (4/7) (IQR 28.6%), with 95 (73.1%) students agreeing or strongly agreeing that instructions at each OSCE station were clear, and an even greater proportion felt the OSCE was well organised (*n* = 114, 87.7%). Despite this, the majority of students still found the OSCE to be intimidating (39.2% agreed, 24.6% strongly agreed).

For the *Fairness* domain, the median positive experience score was 50.0% (2/4) [IQR 50.0], the lowest score of all domains and statistically significantly lower than for *Preparedness*, *Authenticity* and *Assessment for Learning* (all *p* < 0.001) (Figure 2) domains. For example, just over half of students (*n* = 74, 56.9%) thought the OSCE was an accurate assessment of their communication skills, while just under half (*n* = 58, 47.5%) thought the standardised scoring system was fair.

For the *Authenticity* domain, the median positive experience score was 80.0% (4/5) [IQR 40.0]. The majority of students agreed or strongly agreed with all statements within this domain. The statement with the largest majority agreement (*n* = 111, 85.4%) was that the OSCE is a practical and useful experience for students, however fewer (*n* = 78, 60.0%) agreed/strongly agreed that the setting and context at the OSCE stations felt authentic to clinical practice.

For the final domain, *Assessment for Learning*, the median positive experience score was 83.3% (5/6) [IQR 33.3], the highest score of the domains and statistically significantly higher than all other domains (*p* < 0.001) except for *Authenticity* (80%, *p* = 0.776) (Figure 2). Only 2 students (1.6%) felt that a written examination would be a better assessment of communication skills. Most students felt that the OSCE motivated them to learn communication skills (65.4% agreed, 19.2% strongly agreed) and provided opportunities to learn these skills (57.7% agreed, 10.0% strongly agreed).

### 3.3 Comparison of Experience Scores Among Student Groups

No statistically significant differences in positive experience scores were observed between programme of study (BSc/PG Diploma‐MSc) or gender for any of the domains (Table 3). Younger students (20–24 years, *n* = 86) reported lower positive experiences for *Preparedness* (60.0%, IQR 25.0) than those aged 25–29 years (*n* = 21, *p* = 0.045). The most recent cohort included (2017–18 academic year), had significantly lower positive experience scores for both *Authenticity* (60.0% IQR 80.0) and *Assessment for Learning* (66.7% IQR 33.3) compared to all other cohorts (*p* < 0.001). While the 2012–13 and 2015–16 cohorts had identical positive experience scores for *Fairness* (both 50.0% IQR 25.0, *p* > 0.05), the 2012–13 cohort's score was significantly greater than both 2013–14 (25.0% IQR 38.0) and 2017–18 (25.0% IQR 25.0) (*p* < 0.001 for both). The 2013–14 cohort's median score for *Fairness* was also significantly lower than the 2015–2016 cohort, *p* < 0.001.

**Table 3:** Comparison of thematic scores compared between programme of study, gender, age category and cohort year group of students (*n* = 130).

	Domain positive experience percentage score, median [IQR]									
Characteristic	Preparedness	*p* value	Process & environment	*p* value	Fairness	*p* value	Authenticity	*p* value	Assessment for learning	*p* value
All participants	60 [20.0]	—	57.1 [28.6]	—	50.0 [50.0]	—	80.0 [40.0]	—	83.3 [33.3]	—
Programme of study^a^										
BSc (*n* = 74)	60.0 [25.0]	0.821	57.1 [28.6]	0.918	50.0 [50.0]	0.225	80.0 [45.0]	0.769	83.3 [50.0]	0.058
MSc (*n* = 56)	60.0 [20.0]		57.1 [28.6]		50.0 [50.0]		80.0 [40.0]		83.3 [33.3]	
Gender^a^										
Male (*n* = 13)	60.0 [30.0]	0.859	71.4 [28.6]	0.158	50.0 [75.0]	0.689	80.0 [50.0]	0.811	66.7 [16.7]	0.274
Female (*n* = 117)	60.0 [20.0]		57.1 [28.6]		50.0 [50.0]		80.0 [40.0]		83.3 [33.3]	
Age category^b^										
20–24 years (*n* = 86)	60.0 [25.0]^2^	**0.045**	57.1 [32.1]	0.107	50.0 [50.0]	0.364	80.0 [60.0]	0.729	83.3 [33.3]	0.411
25–29 years (*n* = 21)	80.0 [20.0]^1^		71.4 [42.9]		62.5 [50.0]		80.0 [40.0]		83.3 [33.3]	
30+ years (*n* = 21)	80.0 [30.0]		57.1 [42.9]		50.0 [38.0]		80.0 [40.0]		66.7 [41.7]	
Cohort year group^b^										
2012–13 (*n* = 34)	60.0 [40.0]	0.149	71.4 [32.1]	0.104	50.0 [25.0]^2,4^	**< 0.001**	80.0 [40.0]^4^	**< 0.001**	83.3 [33.3]^4^	**< 0.001**
2013–14 (*n* = 41)	60.0 [20.0]		57.1 [28.6]		25.0 [38.0]^1,3^		100.0 [20.0]^4^		83.3 [33.3]^4^	
2015–16 (*n* = 24)	80.0 [15.0]		57.1 [28.6]		50.0 [25.0]^2^		100.0 [35.0]^4^		83.3 [16.7]^4^	
2017–18 (*n* = 31)	60.0 [40.0]		42.9 [42.9]		25.0 [25.0]^1^		60.0 [80.0]^1,2,3^		66.7 [33.3]^1,2,3^	

^a^ Independent Samples Mann Whitney *U* test.

^b^ Independent Samples Kruskal Wallis test, pairwise significance (Dunn's test *p* < 0.05) denoted with superscript ^1,2,3,4^ indicating significant difference from the first, second, third and fourth (where relevant) subgroup. Pairwise significance values have been Bonferroni‐adjusted for multiple testing.

## 4 Discussion

We aimed to evaluate student dietitians' perceptions and experiences of an OSCE designed to assess communication skills in a large cohort of student dietitians. Our findings indicate that the overwhelming majority of student dietitians held positive views regarding the OSCE organisation and processes, the value of the OSCE for learning, and the relevance of the OSCE for future practice. However, some students found the OSCE intimidating, anxiety‐provoking, and felt poorly prepared for the assessment. These views were consistent across all four cohorts despite adaptations to the assessment in response to student feedback, suggesting other factors may have contributed to their perceptions.

In the present study, student dietitians felt the OSCE reflected the skills taught in the module and that they were well supported by facilitators to prepare for the assessment. Despite this, almost half of students reported that the OSCE required a lot of preparation time and a quarter of students still felt poorly prepared. This contrasts with positive views on the adequacy of preparation in a previous evaluation of OSCEs in dietetic education [13]. One reason for this discrepancy between positive perceptions regarding support for the OSCE despite not feeling prepared for it, may be their relative unfamiliarity of this assessment format. In response to this feedback, in the latter two cohorts, a practice formative OSCE was included in advance of the summative OSCE, however this did not result in improvements in student feelings of preparedness. Practice formative OSCEs are resource‐intensive so how they impact student experience and outcomes should be considered. In a study evaluating repeated OSCEs, student dietitians demonstrated only modest improvements in communication skills over time, with the authors concluding that repeat OSCEs should only be provided for students with borderline competence in communication skills who had the greatest improvement between assessments [31]. These findings suggest that despite efforts to prepare students for OSCE assessments, more effective strategies are needed, which could in part be addressed by the use of technology.

Alternative OSCE preparation methods have been proposed for healthcare students. An evaluation of peer‐led preparation OSCEs where medical students rotated the roles of student, patient, and examiner found that repeated practice in an informal setting led to improved confidence and expected performance, although how this impacted subsequent OSCE performance was not explored [32]. The use of video exemplars enhanced preparation for OSCE in nursing students and was associated with greater clarity on the assessment process and expectations [33]. Further research could explore how best to prepare student dietitians for OSCE assessments, particularly as some students find the format stressful. Low resource strategies such as peer preparation and video exemplars should be considered to enhance preparation for OSCE assessments.

Students were positive regarding the organisation of the OSCE and found the instructions for each OSCE station clear, findings that are consistent with other studies evaluating OSCEs in dietetic education [10, 11, 13, 15]. Despite this, almost two‐thirds of students found the OSCE intimidating with a third reporting that this format provoked more anxiety than other types of assessment. Previous studies also report that student dietitians perceived OSCEs to be stressful [10, 15] with some suggestion that this reduced over the duration of the assessment [15]. A systematic review including nine studies in wider health education populations explored anxiety during OSCE and found that whilst anxiety was not associated with performance, anxiety is higher about undertaking OSCEs than in traditional assessments [34].

Considering anxiety in the student dietitian population more broadly, a study in the USA did not find higher levels of stress, anxiety, and depression among 611 student dietitians than in the general population but a small proportion of respondents reported that self‐imposed expectations were a source of stress [35]. In a systematic review of assessment practices in dietetics, assessment has been demonstrated to elicit an emotional response, with students reporting that anxiety can be overcome with well‐designed assessments [6]. One consideration for future research on experiences of OSCEs is that validated scales should be used to measure stress and anxiety as opposed to single answer questions [34]. Strategies such as the need for adequate preparation highlighted above as well as wellbeing initiatives can be optimised to reduce stress and anxiety in relation to assessments [34]. Given the need to prioritise student dietitians' wellbeing, interventions that positively impact on mental health warrant consideration. A six‐session psychoeducation resilience and wellbeing, delivered at an Australian university was found to be valuable by participants and effective in helping them manage assessment related stress through normalising it and emphasising that it does not necessarily impair performance [36]. While not explicitly explored here, the high stakes nature of the OSCE assessment likely also contributed to feelings of anxiety experienced by students.

The least positive domain in our evaluation was the fairness of the OSCE. Only a small majority of students (57%) ‘agreed’ or ‘strongly agreed’ that the OSCE was an accurate measure of their communication skills; just over a quarter (28%) thought the OSCE minimised their chance of failing; and less than half (48%) agreed that the standardised scoring system was fair.

These findings are in contrast to other studies where over 90% of student dietitians deemed an OSCE to be a fair assessment of their skills [10, 11]. These studies had a similar format to the OSCE in the present study but also included a wider range of clinical skills, such as dietary assessment and anthropometry, alongside communication skills. This may be because the assessment of communication skills is perceived to be more subjective than assessments that can be more objectively quantified (e.g. correct calculation of protein intake or weight loss). This is despite the use of a standardised communication skills assessment form being used in the current OSCE, a strategy which is recommended as best practice [4] and has been positively received by healthcare students in previous OSCE research [37]. The perception of unfairness was consistent across cohorts even when the assessment changed to a hurdle assessment from a format where marks were awarded suggesting that educators could consider assessing these skills formatively rather than summatively.

Perceptions of the OSCE as an unfair assessment have been reported in OSCE evaluations in other healthcare students. Dental students deemed it unfair to be assessed on skills that they had only recently been taught and had limited opportunities to practice [38]. This may be a factor for the students in the present study as the OSCE assessment occurs at the end of a 12‐week module, at which time the students had very limited practice placement experience. Other factors that have been reported to impact on perceptions of fairness in OSCEs in speech and language therapy students include a lack of inter‐rater reliability between different assessors and a view that some assessors may be more intimidating and mark more harshly [39]. Ensuring robust training of assessors should be prioritised [4]. Qualitative approaches to future research such as focus groups may yield more in‐depth insights as to why some student dietitians perceive OSCEs to be an unfair assessment.

The majority of students were positive about the authenticity of the OSCE, finding it an accurate assessment of the communication skills required for dietetics; a useful experience; good preparation for placement and reporting that the context felt faithful to clinical practice. Previous studies have reported challenges to achieving authenticity in OSCEs due to the fragmentation of tasks into individual clinical skills, time pressure, variable portrayal of patient cases by different simulated patients and the interaction between assessors, patients and students [40] In our model, simulated patients receive training, cases were developed in collaboration between dietitians in academia and clinical practice, and as the OSCE assesses only communication skills it requires a holistic approach to a patient consultation rather than fragmentation of an individual clinical tasks. Since OSCEs are simulated experiences, students' prior exposure to simulation may influence their perceptions of OSCEs. In a scoping review of communication skills teaching and assessment in dietetics, 13 of 45 studies used simulation‐based approaches, highlighting their common use in dietetic education [22].

The majority of student dietitians reported positive views of the OSCE as a learning experience, including that the OSCE provided an opportunity to learn and motivated them to learn communication skills. This is consistent with other OSCE evaluations in dietetic education and is an encouraging finding as there is evidence that OSCE performance is linked to practice placement outcome [10, 11]. This association points towards OSCEs playing an important role in programmatic assessment in dietetic education where holistic judgements of competence are required [41]. In a study including 328 students who completed an OSCE, there was a significant positive relationship between the marks awarded for some OSCE stations (active stations including assessment of communication skills) and the authors proposed that OSCEs may have a role in identifying students requiring additional support before practice placement [10]. Effective feedback is integral to learning from the OSCE but less than half of students in the present study agreed that feedback was helpful. This may be associated with operational issues such as timing of feedback (which may have occurred after the survey questionnaire was administered) or may represent wider dissatisfaction with the feedback provided. Studies have shown that healthcare students value detailed individualised feedback over just a mark following an OSCE: feedback can be written, face‐to‐face or audio‐recorded but it can be challenging for assessors to provide highly detailed feedback in the time available [42]. A previous study reported that students value OSCEs as a formative assessment with feedback being used to improve practice but find summative OSCEs inauthentic, lacking links to what is taught and with limited feedback [43].

In our study, despite minor adjustments made to the OSCE format over the academic cohorts in response to student and assessor feedback, consistent domains emerged regarding students' perceptions and experiences of the OSCEs. A notable difference in experiences, was that younger students reported less positive experiences for preparedness. One‐third of participants were over 25 years old at the time of the assessment and research has suggested that older students may be better equipped for assessments due to prior study experiences and a desire to do well [44]. Other characteristics related to being older, such as workplace communication experience due to prior work roles, may specifically influence feelings of preparation for a communication skills OSCE assessment.

This study has demonstrated widespread positive perceptions and experiences of OSCEs among student dietitians. Despite the positive experiences reported here and the positive impacts on dietetic education, disadvantages of OSCEs include the intensive resources required, such as time, staffing for assessors and patient actors, as well as physical resources of simulated clinical environments. In view of this, alternative assessment formats have been considered. For example, a study in 169 student dietitians in Australia found oral interviews used less resources and were able to predict placement outcome [7] and virtual OSCEs have been explored in health professions education particularly as telehealth becomes a more prominent feature of clinical practice [45]. Technological advances have provided alternatives to traditional approaches for teaching communication skills with artificial intelligence and machine learning being used to simulate virtual patients that learners can interact with [46]. Furthermore, extended reality offers the learner the opportunity to be immersed in a virtual scenario interacting with avatars through head mounted displays or goggles [47]. Artificial intelligence has the potential to create more diverse virtual patient scenarios than traditional simulated patients and has been effective in the development and monitoring of communication skills in healthcare [48]. As technology continues to advance, educators could consider moving away from traditional teaching and assessment methods, however aspects like user interface (e.g. via text alone compared to fully immersive artificial reality) must be evaluated and tailored to the educational aim of the activity.

### 4.1 Strengths, Limitations and Future Research

The strengths of this study are the large number of respondents for a small discipline such as dietetics, the high response rate from eligible participants and that it provides an in‐depth understanding of student dietitians' perception of an OSCE across four academic years. The variations across cohorts including the number of stations, evolving case content, changing pool of assessors and standardised patients may have influenced students' experiences and perceptions and this should be considered in interpreting the results. However, evaluating student cohorts of multiple years is a common feature of action research in education and a necessary compromise to recruit large and representative cohorts of students from a traditionally small profession. Changes in practice, curriculum content and student demographics since the inception of the study could subtlety influence the findings and whilst the study provides a rich longitudinal data set with consistent observations, these factors should be considered. The evaluation used an unvalidated questionnaire to collect perceptions and experiences, and formal development and evaluation of such a questionnaire in the future is recommended. A further limitation is that whilst the question format captures data on the positive and negative aspects of students' experiences, it does not allow for in‐depth understanding of the factors influencing their views. To better understand students' experiences and learning through engaging with OSCEs, qualitative approaches should be considered for future research [49]. Future research could also consider student dietitians' evaluation of alternative assessment formats such as objective structured video assessments [50], portfolio assessment to demonstrate skill progression [51], oral assessments [7] or artificial intelligence [48].

In conclusion, the findings of this study suggest that an OSCE to assess communication skills is a valuable learning experience for student dietitians but that further consideration needs to be given to preparation, tools for assessment, and feedback.

## Author Contributions

**Annemarie Knight:** Data curation (equal); formal analysis (equal); project administration (lead); writing – original draft (lead); **Kevin Walsh:** Data curation (lead); formal analysis (lead); visualisation (lead); project administration (equal); writing – review & editing (equal); **Dianne P. Reidlinger:** Conceptualization (lead); investigation (lead); methodology (lead); project administration (equal); writing – review & editing (supporting): **Patrica Thomas‐Owolabi:** Data curation (supporting); Formal analysis (supporting); writing – review & editing (supporting); **Kevin Whelan:** Conceptualization (lead); data curation (equal); formal analysis (equal); investigation (lead); methodology (lead); project administration (lead); supervision (lead); writing – original draft (equal).

## Ethics Statement

The authors have nothing to report.

## Conflicts of Interest

The authors declare no conflicts of interest.

## Transparency Declaration

The authors affirm that this manuscript is an honest, accurate, and transparent account of the study reported, and that no important aspects have been omitted and that any discrepancies from the study as planned have been explained.

## Data Availability

The authors have nothing to report.
